# Modified Whole-Mount *In situ* Hybridization Protocol for the Detection of Transgene Expression in Electroporated Chick Embryos

**DOI:** 10.1371/journal.pone.0002638

**Published:** 2008-07-09

**Authors:** Natacha Arede, Ana T. Tavares

**Affiliations:** Instituto Gulbenkian de Ciência, Oeiras, Portugal; University of Giessen Lung Center, Germany

## Abstract

**Background:**

*In vivo* electroporation has been extensively used as an effective means of DNA transfer for analyzing gene function as well as gene regulation in developmental systems. In any of these two types of studies, the correct spatial and temporal expression of the electroporated transgene can only be accurately assessed by *in situ* hybridization.

**Methodology/Principal Findings:**

While analyzing transgene expression in electroporated chicken embryos, we verified that transgene riboprobes cross-hybridized with the exogenous plasmid DNA when embryos were processed by conventional whole-mount *in situ* hybridization (WISH).

**Conclusions/Significance:**

Here we describe a modification to the WISH protocol that is essential to prevent DNA cross-hybridization and to specifically detect transgene mRNA transcripts in electroporated embryos. Our optimized WISH procedure can be applied not only to electroporated chick embryos but also to other embryos or adult tissues that have been transfected with large amounts of reporter- or expression construct DNA.

## Introduction

*In vivo* electroporation is a very effective technique for introducing DNA into cells of various animal models, including *Drosophila* [1], ascidians [2], zebrafish [3], axolotl [4], *Xenopus* [5], chick (reviewed in [6]) and mouse [7]. This method makes use of electric pulses to create transient pores in the plasma membrane through which the negatively charged DNA molecules enter the cell. The electroporation of living embryos has been extensively used in gain-of-function studies, for which the expression vectors carry a ubiquitous promoter driving the transgene transcription, as well as in loss-of-function approaches, such as those using siRNA or morpholinos (reviewed in [6]). In addition to gene function, the electroporation method has also emerged as a powerful tool to analyze gene regulatory sequences, particularly in chicken (reviewed in [8], [9]) and ascidians [2] embryos.

In most functional studies, the expression construct is co-electroporated with a fluorescent reporter plasmid to evaluate the efficiency of transfection in live tissues or embryos. Since both vectors carry the same regulatory sequences, reporter fluorescence is also used to indirectly monitor the expression of the exogenous gene. However, the correct transcription of the transgene can only be accurately assessed by *in situ* hybridization. Fluorescent reporters are also broadly used as readout of enhancer activity in studies of gene regulation. However, fluorescence becomes visible at least two to three hours after induction of transcription. Therefore, to determine exactly when and where a certain enhancer is active, reporter mRNA localization must be investigated. Here we describe a modification of the conventional whole-mount in situ hybridization (WISH) procedure that proved to be crucial for the correct detection of transgene transcripts in electroporated embryos.

## Results

**Figure 1:**
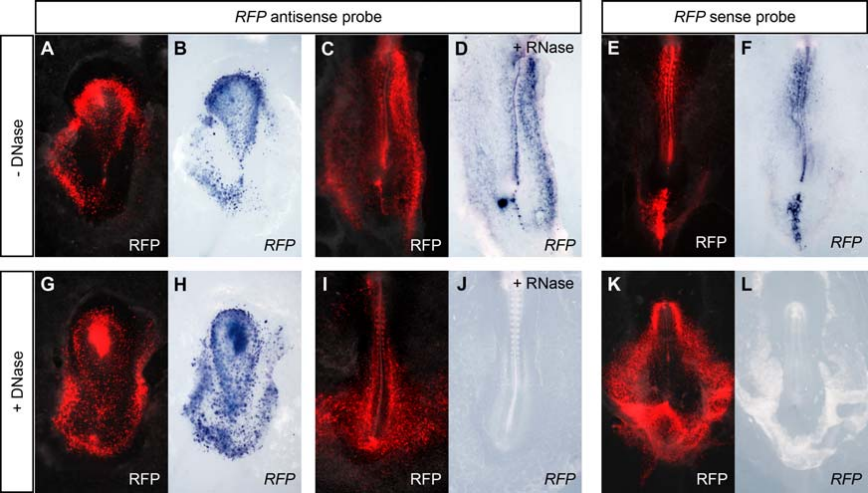
Comparison between RFP fluorescence and *RFP* expression patterns in CAGGS-RFP electroporated chick embryos. Chick embryos were electroporated with the ubiquitous reporter construct pCAGGS-RFP and processed for WISH. (A, C, E, G, I, K) Merge of bright field with RFP fluorescence images. (B, D, F, H, J, L) Detection of *RFP* antisense and sense probes by WISH. *RFP* antisense probe was detected in all RFP-fluorescent cells of embryos processed by conventional WISH (A, B). A similar co-localization was observed in embryos either treated with RNase A prior to hybridization (D) or hybridized with the *RFP* sense probe (F). When embryos were treated with DNase I, the antisense probe was also detected in the *RFP*-expressing cell population (H), but not in RNase A-treated embryos (J), and the sense probe was undetectable (L).

In order to investigate the transcription pattern of an overexpressed transgene, chick embryos were electroporated with the pCAGGS-RFP construct, which carries the cDNA of monomeric red fluorescent protein (RFP; [10]) under the control of the CAGGS ubiquitous promoter [11], and the pattern of fluorescence was compared with the localization of *RFP* transcripts. When embryos were processed by conventional whole-mount *in situ* hybridization (WISH; [12], [13]), the *RFP* antisense riboprobe was detected in the red fluorescent cells (Figure 1A,B). However, a similar pattern was seen not only in embryos treated with RNase A before hybridization (Figure 1C,D) but also in those hybridized with the *RFP* sense probe (Figure 1E,F). The digestion of RNA transcripts with RNase A as well as the hybridization of the sense probe are expected to work as controls for background staining. These observations suggested that the RFP probes were hybridizing with the electroporated DNA of the reporter construct. This DNA cross-hybridization was transgene-specific and was not observed when transgene-unrelated probes were used, such as those against *eGFP* (data not shown) and left-side specific genes [14].

To avoid cross-hybridization, we tested different enzymatic digestions and stringency conditions. We could eliminate the detection of electroporated DNA in three situations: (i) DNase I digestion before hybridization, to degrade the electroporated DNA, (ii) RNase H treatment after hybridization, to eliminate the DNA-RNA hybrids (Figure S1A–D), and (iii) probe hybridization at 55°C, to avoid DNA denaturation (Figure S1E–H). Among them, the first condition proved less disturbing to the embryo integrity and to cause less background. After DNase treatment, the RFP antisense probe still labeled the *RFP*-expressing cell population (Figure 1G,H), but not in embryos digested also with RNase A (Figure 1I,J), whereas the RFP sense probe was no longer detected (Figure 1K,L). These results indicate that DNase digestion is essential to avoid DNA cross-hybridization and to exclusively detect transgene transcripts in embryos electroporated with expression constructs.

**Figure 2:**
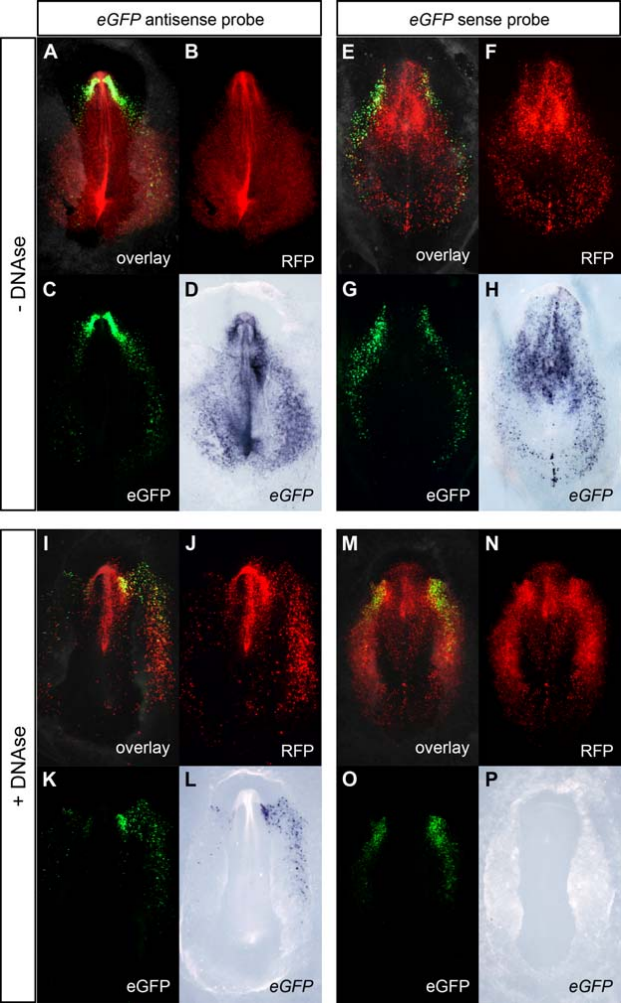
Comparison between eGFP fluorescence and *eGFP* expression patterns in Cer0.4-eGFP/CAGGS-RFP electroporated chick embryos. Chick embryos were co-electroporated with Cer0.4-eGFP (tissue-specific reporter) and pCAGGS-RFP (ubiquitous reporter) at stage HH3 and processed for WISH. (A, E, I, M) Merge of bright field with fluorescence images. (B, F, J, N) RFP fluorescence. (C, G, K, O) eGFP fluorescence. (D, H, L, P) WISH using *eGFP* antisense and sense probes. At stages HH6-7, RFP fluorescence was detected in all electroporated cells, whereas eGFP fluorescence was specifically observed in the anterior mesendoderm. When embryos were processed by the standard WISH method, the *eGFP* antisense probe was detected in both eGFP- and RFP-positive cells (D). These cells were also labeled by the *eGFP* sense probe (H). In embryos treated with DNase I, the antisense probe was restricted to the *eGFP*-expressing cell population (L), whereas the sense probe was no longer detected (P).

**Figure 3:**
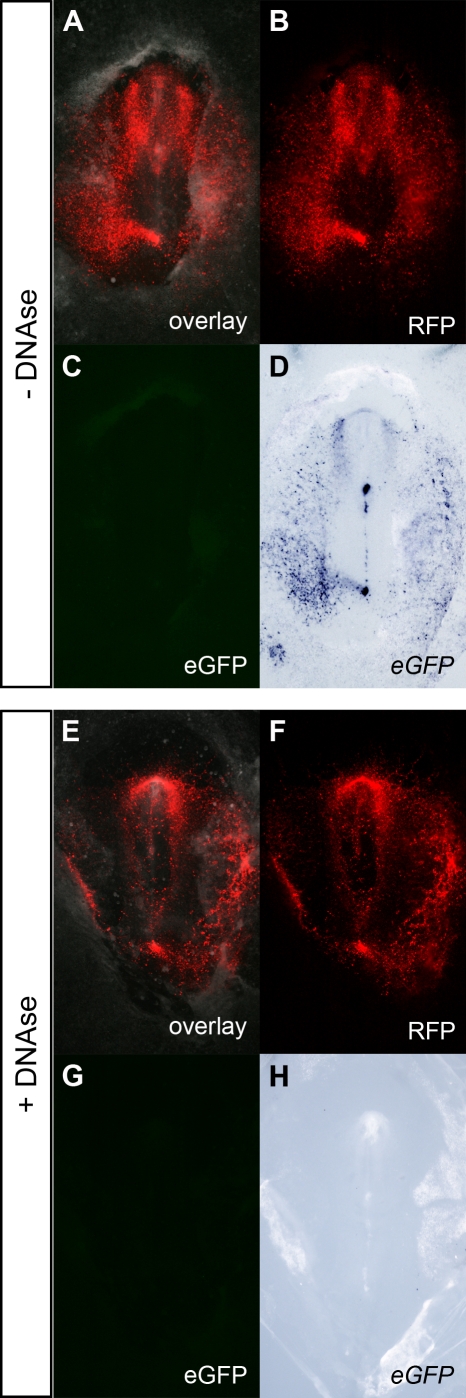
Comparison between eGFP fluorescence and *eGFP* expression patterns in Cer0.12-eGFP/CAGGS-RFP electroporated chick embryos. Chick embryos were co-electroporated with Cer0.12-eGFP (silent reporter) and pCAGGS-RFP (ubiquitous reporter) at stage HH3 and processed for WISH. (A, E) Merge of bright field with fluorescence images. (B, F) RFP fluorescence. (C, G) eGFP fluorescence. (D, H) WISH using the *eGFP* antisense probe. At stage HH6, RFP fluorescence was observed throughout the electroporated embryos, whereas eGFP fluorescence was undetectable. After WISH, the *eGFP* probe was detected in untreated embryos (D) but not in DNase I-treated embryos (H).

The modification of the WISH protocol proved to be indispensable in gene regulation studies using tissue-specific reporters. During our study of the transcriptional regulation of chick *Cerberus* (*cCer*) in early development, *cCer* 5′ genomic fragments were subcloned upstream the enhanced green fluorescent protein (eGFP) and cCer-eGFP constructs were introduced into chick embryos by electroporation [14]. The ubiquitous reporter pCAGGS-RFP was co-electroporated to label the populations of targeted cells. In embryos electroporated with the Cer0.4-eGFP reporter, which carries the complete regulatory region of the *cCer* gene (i.e., the 400 base pairs sequence upstream the ATG; [14]), eGFP fluorescence was restricted to the anterior mesendoderm (Figure 2). However, when these embryos were processed by standard procedures for WISH, the *eGFP* antisense probe labeled not only the *eGFP*-expressing cells but also the RFP-positive cells (Figure 2A–D). The electroporated cells were also labeled by the *eGFP* sense probe (Figure 2E–H), indicating once again that both probes were cross-hybridizing with the DNA of the eGFP reporter construct. The detection of plasmid DNA was eliminated in embryos treated with DNase I before probe hybridization: the *eGFP* antisense probe specifically labeled the eGFP fluorescent cells (Figure 2I–L), whereas the *eGFP* sense probe was no longer detected (Figure 2M–P). These observations demonstrate that the addition of a DNase digestion step to the WISH protocol is fundamental for the correct localization of tissue-specific reporter transcripts in enhancer studies. Our modified WISH procedure was particularly important for the expression analysis of silent reporter constructs, such as Cer0.12-eGFP, which carries the minimal promoter of the *cCer* gene (i.e., the 120 base pairs sequence upstream the ATG; [14]). In embryos co-electroporated with Cer0.12-eGFP and pCAGGS-RFP, eGFP fluorescence was undetectable (Figure 3). However, the *eGFP* antisense probe was detected in the *RFP*-expressing cell population when embryos were processed by conventional WISH (Figure 3A–D). The absence of *eGFP* expression was revealed only in embryos treated with DNase I (Figure 3E–H).

In summary, our observations suggest that the mRNA expression of electroporated transgenes can only be accurately assessed if a DNase step is added to the standard WISH protocol. This modified procedure is especially crucial in reporter- our expression assays using tissue-specific enhancers.

## Discussion

We have shown that, when electroporated embryos are processed by conventional WISH techniques for the detection of transgene expression, transgene riboprobes hybridize not only with the transgene mRNA transcripts but also with the plasmid DNA. One of the reasons for this cross-hybridization is the fact that the electroporated DNA is delivered in very large amounts (i.e., 100–200 nanograms per embryo) and can remain in cell nuclei for many days [15]. In contrast, in transgenic zebrafish, *Xenopus* or mouse embryos, transgene copies are much fewer and undetectable by standard WISH protocols (e.g. [14]). DNA cross-hybridization may also be triggered by the stringency conditions generally used for WISH (i.e., hybridization at 70°C in 50% formamide), which can promote the denaturation of the electroporated plasmid DNA [16] and its hybridization with complementary riboprobes [17], [18]. Indeed, we observed that reducing the hybridization temperature to 55°C was enough to avoid DNA cross-hybridization and confer specificity to the detection of *eGFP* transcripts in electroporated embryos (see Figure S1E–H).

The detection of transgene transcripts by WISH in electroporated embryos has been reported in studies using ubiquitous expression vectors (e.g. [3], [8]). In these type of experiments, since the transgene is transcribed in all targeted cells, its expression pattern coincides with the distribution of the plasmid DNA and, therefore, the hybridization of transgene probes with the electroporated DNA is imperceptible (see Figure 1). DNA cross-hybridization becomes most evident in electroporation studies using tissue-specific enhancers (Figures 2 and 3). In any of these two types of assays, we have demonstrated that the binding of transgene probes to the electroporated DNA can be avoided when the embryos are treated with DNase I before hybridization.

In conclusion, we describe an optimized protocol for WISH that is crucial for the accurate detection of transgene expression in electroporated embryos. In addition to chick embryos, this modified procedure is applicable to other embryos and to adult tissues, such as those subject to gene therapy by electroporation [19]. Moreover, our WISH procedure may provide a reliable way to localize transgene expression whenever large amounts of naked plasmid DNA are transferred into tissues, not only by electroporation but also by other gene delivery methods.

## Materials and Methods

### Embryo electroporation

Fertilized chicken eggs were purchased from Quinta da Freiria (Bombarral, Portugal) and incubated at 37.5 degrees C for the appropriate period. Embryos were staged according to Hamburger and Hamilton (HH; [20]) and processed as described by Tavares et al. [14]. In brief, embryos were explanted at stages HH3-5, injected with plasmid DNA solution (2 mg/ml of Cer-eGFP constructs; 0.5 mg/ml of the pCAGGS-RFP construct; 0.1% Fast Green; Sigma-Aldrich), and electroporated using 2-mm square electrodes (CY700-1Y and CY700-2; Nepa Gene) and a square wave electroporator (ECM830; BTX). Embryos were then placed in New culture [21], incubated at 37°C until stages HH6-10 and photographed under a fluorescence stereomicroscope (Leica MZ16FA).

### Whole-mount *in situ* hybridization

Embryos were fixed overnight in 4% paraformaldehyde (PFA) in phosphate-buffered saline (PBS) plus 0.1% Tween® 20 (PBT) at 4°C, dehydrated though a series of methanol/PBT solutions (25%, 50%, 75% and 100% methanol), and stored at −20°C until hybridization. Fixed embryos were rehydrated and rinsed twice in PBT. At this point, embryos were either digested with DNase and/or RNase, or kept in PBT. For DNA digestion, embryos were incubated with RNase-free DNase I (50 U/ml in DNase I buffer; Ambion) for 1h at 37°C. For the elimination of RNA transcripts, embryos were treated with DNase-free RNase A (100 μg/ml in PBT) for 1h at 37°C. The RNase A enzyme was inactivated with 0.5x standard saline citrate (SSC)/0.1%SDS for 10 min at room temperature.

All embryos were bleached in 6% hydrogen peroxide in PBT for 1h. Embryos were then rinsed 3 times in PBT for 5 min, digested with proteinase K (10 μg/ml in PBT) for 5 min at room temperature, washed once in 2 mg/ml glycine in PBT and twice in PBT for 5 min each, and post-fixed in 4% PFA/0.2% glutaraldehyde in PBT for 20 min at room temperature. Embryos were subsequently rinsed twice in PBT for 5 min and pre-hybridized at 70°C in hybridization solution (50% formamide, 5x SSC, pH 5, 0.1% Tween® 20, 50 μg/ml heparin, 50 μg/ml *Torula* RNA, 50 μg/ml salmon sperm DNA) for 2h. Embryos were then incubated overnight at 70°C in hybridization solution containing 500 ng/ml of denatured riboprobe. Riboprobes were generated by *in vitro* transcription in the presence of Digoxigenin-UTP (Roche Diagnostics). The antisense and sense probes span their entire coding sequences of *eGFP* and *RFP* and were synthesized from linearized pCS2-eGFP and pCAGGS-RFP plasmids, respectively. On the second day, embryos were washed twice in 50% formamide/4x SSC, pH 5/1% SDS and twice in 50% formamide/2x SSC, pH 5 for 30 min each. These post-hybridization washes were carried out at 55°C. Embryos were then rinsed three times for 5 min in MABT (100 mM maleic acid, 150 mM NaCl, pH 7.5, 0.1% Tween®), blocked for 2 h at room temperature in 10 % goat serum in MABT, and incubated overnight at 4°C in 1% goat serum in MABT with 1∶5000 alkaline phosphatase-coupled anti-Digoxigenin antibody (Roche Diagnostics). On the third day, embryos were washed in MABT twice for 5 min and five more times for 1 h each. Embryos were then rinsed twice in NTMT (100 mM NaCl, 100 mM TrisHCl, pH 9.5, 50 mM MgCl2, 0.1% Tween®) for 15 min each, followed by the staining reaction in BM Purple (Roche Diagnostics) in the dark for 30 min to 12 h. Stained embryos were fixed overnight in 4% PFA in PBT, stored in PBT and photographed under a Leica MZ16FA stereomicroscope.

## Supporting Information

Figure S1 (2.36 MB PDF)
