# Supplementary material for: Modified Whole-Mount In situ Hybridization Protocol for the Detection of Transgene Expression in Electroporated Chick Embryos
Source: PLoS One. 2008 Jul 9;3(7):e2638. doi: 10.1371/journal.pone.0002638 (PMC2441435; doi:10.1371/journal.pone.0002638)
Supplement: Figure S1 — (2.36 MB PDF) [file pone.0002638.s001.pdf]

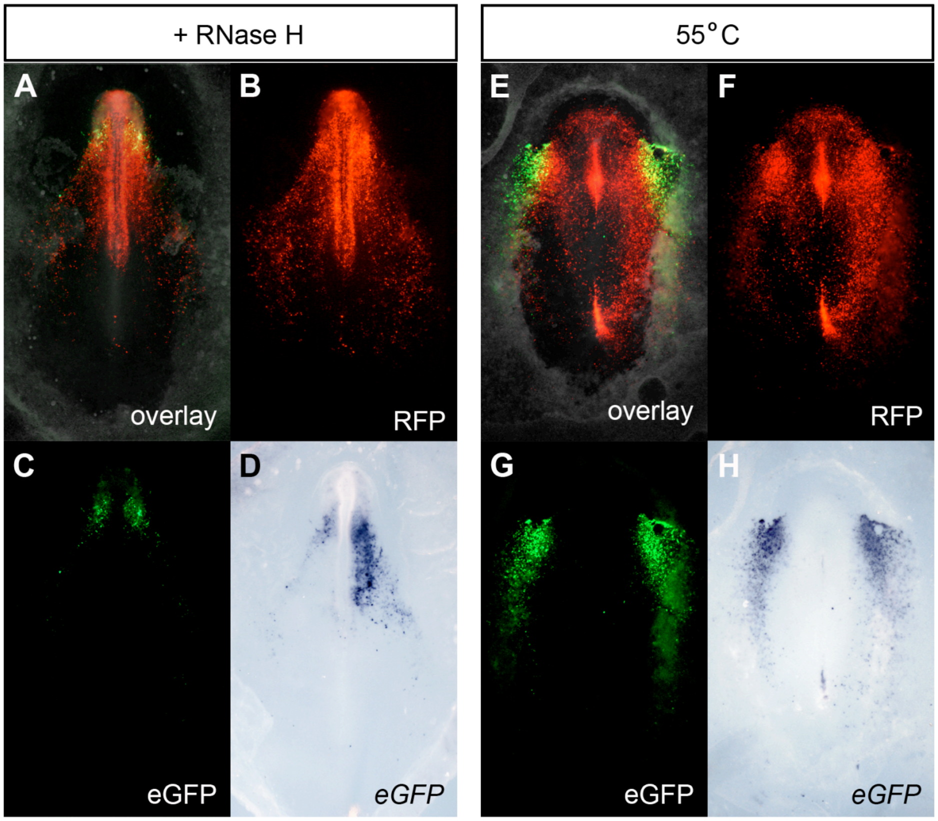

Figure S1

Additional modifications of the WISH protocol that prevent DNA cross-hybridization of the *eGFP* probe.

Chick embryos were co-electroporated with Cer0.4-eGFP and pCAGGS-RFP (ubiquitous reporter) and processed for WISH. (A, E) Merge of bright field with fluorescence images. (B, F) RFP fluorescence. (C, G) eGFP fluorescence. (D, H) WISH using the *eGFP* antisense probe. (D) After the post-hybridization washes, embryos were treated with RNase H (Ambion; 8 U/ml in 75 mM KCl, 50 mM Tris-HCl, pH 8.3, 3 mM MgCl2) for 1h at 37° C. (H) The pre-hybridization and hybridization steps of the WISH protocol were performed at 55° C.

At stages HH6-7, RFP fluorescence was detected in all electroporated cells (B, F), whereas eGFP fluorescence was specifically observed in the anterior mesendoderm (C, G). When embryos were treated with RNase H (D) or hybridized at 55° C (H), the *eGFP* antisense probe labeled only the *eGFP*-expressing cells. At stage 7, *eGFP* transcripts start to be detected also in the left paraxial and lateral plate mesoderm (D; see [14]).
